# Mitigating the Growth, Biochemical Changes, Genotoxic and Pathological Effects of Copper Toxicity in Broiler Chickens by Supplementing Vitamins C and E

**DOI:** 10.3390/ani11061811

**Published:** 2021-06-17

**Authors:** Mohamed A. Hashem, Sahar S. Abd El Hamied, Eman M. A. Ahmed, Shimaa A. Amer, Mohamed E. El-Sharnouby

**Affiliations:** 1 Clinical Pathology Department, Faculty of Veterinary Medicine, Zagazig University, Zagazig 44511, Egypt; mhashem.vet@gmail.com; 2 Clinical Pathology Department, Animal Health Institute, Zagazig Branch, Zagazig 44511, Egypt; sahar_elmasry91@yahoo.com (S.S.A.E.H.); emanalilogy@gmail.com (E.M.A.A.); 3 Department of Nutrition & Clinical Nutrition, Faculty of Veterinary Medicine, Zagazig University, Zagazig 44511, Egypt; 4 Department of Biotechnology, College of Science, Taif University, P.O. Box 11099, Taif 21944, Saudi Arabia; m.sharnouby@Tu.edu.sa

**Keywords:** copper toxicity, antioxidants, liver function tests, liver histoarchitecture, lipid profile

## Abstract

**Simple Summary:**

Copper (Cu) is a trace element necessary for biological utility; nevertheless, it can produce significant harmful impacts when existing in abundance. This study examined the efficiency of vitamin C and vitamin E in alleviating the biochemical, genotoxicity, and pathological alterations in the liver induced by copper sulfate (CuSO_4_) toxicity in chickens. The broilers were fed on five experimental diets; basal diet with no additives or basal diets supplemented with 300 mg CuSO_4_/kg, CuSO_4_ + 250 mg Vit. C/kg diet, CuSO_4_ + 250 mg Vit. E/kg diet, CuSO_4_ + 250 mg Vit. C/kg diet + 250 mg Vit. E/kg diet for six weeks. The obtained results suggested that addition of vitamin C and E, especially in combination, was beneficial for alleviating the harmful effects of CuSO_4_ toxicity on growth performance and liver histoarchitecture in broiler chickens.

**Abstract:**

This experiment was carried out to explore the efficiency of an individual or combined doses of vitamin C (Vit. C) and vitamin E (Vit. E) in alleviating biochemical, genotoxicity, and pathological changes in the liver induced by copper sulfate (CuSO_4_) toxicity in broiler chickens. Two hundred and fifty-one-day-old broiler chicks were haphazardly allotted into five groups (five replicates/group, ten chicks/replicate). The birds were fed five experimental diets; (1) basal diet with no additives (CON), (2) basal diets supplemented with 300 mg CuSO_4_/kg diet (CuSO_4_), (3) basal diets supplemented with 300 mg CuSO_4_/kg diet + 250 mg Vit. C /kg diet, (4) basal diets supplemented with 300 mg CuSO_4_/kg diet +250 mg Vit. E /kg diet, (5) basal diets supplemented with 300 mg CuSO_4_/kg diet + 250 mg Vit. C /kg diet + 250 mg Vit. E /kg diet for six weeks. The results displayed that CuSO_4_-intoxicated birds had significantly (*p* < 0.05) decreased bodyweight, weight gain, and feed intake with increased feed conversion ratio from the 2nd week till the 6th week compared with the CON. However, these changes were minimized by single or combined supplementation of vitamin C and E. The FCR was insignificantly different in birds-fed diets complemented with vitamin C and E singly or in combination from the 3rd week of age compared to the CON. Serum aminotransferases (ALT, AST) and alkaline phosphatase (ALP) were elevated in CuSO_4_-intoxicated birds (*p* < 0.05). Additionally, they showed a drop in serum total protein (TP), albumin, globulins, triglycerides (TG), total cholesterol (TC), low-density lipoprotein-cholesterol (LDL-C), very low-density lipoprotein-cholesterol (VLDL-C), and high-density lipoprotein-cholesterol (HDL-C) levels compared to the CON (*p* < 0.05). Concomitantly, histopathological and DNA changes were perceived in the liver of CuSO_4_-intoxicated birds. Co-supplementation of Vit. C and Vit. E single-handedly or combined with CuSO_4_-intoxicated chickens enhanced the performance traits and abovementioned changes, especially with those given combinations of vitamins. From the extant inquiry, it could be established that supplementation of vitamin C and E was beneficial for mitigating the harmful effects of CuSO_4_ toxicity on growth performance and liver histoarchitecture in broiler chickens.

## 1. Introduction

The proper activities of iron metabolism-related metalloenzymes are maintained by copper (Cu), a vital microelement involved in poultry diets [1]. Despite the need for unlike enzyme activities and metabolic processes, chronic overexposure to copper caused adverse effects [2]. This leads to cell death because of its ability to stimulate the production of Reactive Oxygen Species (ROS) [3]. Copper has a narrow optimum range between elemental and toxic concentrations [4]. Naturally, some types of soil contain Cu in toxic levels. In contrast, others may comprise high Cu levels through the human release of heavy metals into the environment through melting, mining, farming, industrial, and waste removal practices [5]. In poultry, the extreme type of Cu poisoning is the long-term ingestion of Cu compounds from different sources [6]. Copper metabolism, its release into the circulatory system or excretion through the bile, is controlled primarily by the liver [7]. It accumulates steadily in the liver during chronic Cu toxicity without causing any noticeable signs or symptoms. When the liver storage capacity is surpassed, it can lead to hepatocellular lesions and the Cu releases into the blood circulation causing jaundice, hemolysis, and renal disease [8]. The studies mentioned above have designated that excessive exposure to Cu can cause oxidative stress in the brain tissue of chickens [9], reduce the glutathione peroxidase and copper-zinc superoxide dismutase activities, and increase the contents of hydroxyl radical and malondialdehyde in the liver of ducklings [10]. A discrepancy between production of ROS and the ability of the body to detoxify these intermediate species is indicative of oxidative stress. Wang et al. [4] showed that Cu encourages oxidative damage in skeletal muscles of chickens through autophagy, apoptosis, and mitochondrial dynamics, thus expressing fears about poultry raising zones polluted with Cu. Moreover, Wang et al. [11] reported nephrotoxicity in Cu-intoxicated chickens due to oxidative damage of the kidney. Although Cu supplementation of up to 200 ppm is necessary to promote growth, excessive amounts of dietary Cu reduce growth. It reduces the digestibility and absorption of copper in poultry, which leads to increased excretion in the faeces and environmental pollution. [12].

Few reports have displayed that supplementation of antioxidants such as vitamin C vitamin E, polyphenols, alpha-lipoic acid, beta-carotene, and zinc has a protective impact against the toxicity of Cu [13,14]. However, only restricted studies have been achieved on the effects of vitamin C and E single-handedly or incorporating on growth performance, biochemical markers, DNA damage, or pathological findings in broilers fed excess dietary Cu. Consequently, the ambition of the existent inquest was to assess the effects of Cu intoxication on these parameters and then appraise the protecting impact of vitamins C and E against excess dietary supplementation with Cu, individually or in combination. Therefore, this experiment aimed to assess the mitigating effects of single or combined addition of vitamin C and E on the harmful impacts of Cu toxicity in broiler chickens.

## 2. Material and Methods

### 2.1. Experimental Birds, Diet, and Protocol

This study was conducted in a poultry research unit in the faculty of veterinary medicine, Zagazig University, Egypt. The ethics of the experimental protocol were approved by the Institutional Animal Care and Use Committee of Zagazig University, Egypt (ZU-IACUC/2020). All animal experiments were performed following the recommendations described in “The Guide for the Care and Use of Laboratory Animals in scientific investigations.”

Two hundred and fifty-one-day-old commercial broiler chickens (COBB-500) were attained from Al-Kahira Poultry Company, 10th of Ramadan City, Sharkia Governorate, Egypt. The experiment lasted for 42 days with good ventilation. Birds were raised in an open, well-ventilated house with sawdust. Room temperature was controlled and thermostatically regulated by two heaters. Room temperature during the first week was set at 34 °C and gradually reduced by 3 °C every week until it reached 24 °C. The light program for the first week was 24 hours a day and then changed to 16 h of light and 8 hours of dark over 7 to 42 days.

Freshwater and feed were offered for *ad libitum* consumption throughout the experiment. The chicks were given a starter diet from one day until the 10th day of age, a grower diet (11th–22nd day), followed by a finisher diet up to 42-days of age. Ingredients and chemical composition of diets were formulated as designated by the COBB-500 broiler manual guide [15] (Table 1). All birds were vaccinated at 7 and 14 days old against Newcastle disease and 11 and 22 days old for Gumboro disease [16].

The chicks were haphazardly allotted into five experimental groups (five replicates/group, 10 chicks/replicates). The birds were fed on five experimental diets; (1) basal diet with no additives (CON), (2) basal diets supplemented with 300 mg CuSO_4_/kg diet (CuSO_4_), (3) basal diets supplemented with 300 mg CuSO_4_/kg diet + 250 mg Vit. C /kg diet, (4) basal diets supplemented with 300 mg CuSO_4_/kg diet + 250 mg Vit. E /kg diet, (5) basal diets supplemented with 300 mg CuSO_4_/kg diet + 250 mg Vit. C/kg diet + 250 mg Vit. E/kg diet for six weeks. Copper sulfate (CuSO_4_·5H_2_O, El-Gomhoria industry, Zagazig, Egypt), vitamin C (ROVIMIX^®^ STAY-C^®^35, DSM, Heerlen, The Netherlands), and vitamin E (α tocopherol acetate, Pharco Pharmaceutical Industries, Zagazig, Egypt). The toxic dose of Cu used in this inquest was dogged according to Cinar et al. [17], while the vitamins C and E doses were used after Sahin et al. [18].

### 2.2. Growth Performance

The average initial body weight (BW) was recorded at the beginning of the experiment. The BW was then determined every week, and body weight gain (BWG) was determined [19]. The difference between the weight of the provided feed and the feed that remained was used to calculate feed intake (FI) per replicate. Then, the feed conversion ratio (FCR) was calculated. FCR = amount of consumed feed (g)/BWG (g).

### 2.3. Sampling

Samples of blood were collected from the wing vein of 10 randomly selected birds in each group at the termination of 3rd and 6th weeks post-supplementations and centrifuged (3000 rpm for 15 min) immediately for separation of serum, which is stored at −20 °C in deep freeze until biochemical analysis [20]. The chicks were euthanized using cervical dislocation, according to the American Veterinary Medical Association (Schaumburg, IL, USA) guidelines [21], and two portions of liver tissues were separated and blotted dry. The first part was put in ice-cold PBS (phosphate buffer saline) for comet assay determination, and the 2nd part was fixed in ten percent formalin for histopathological inspection.

### 2.4. Blood Biochemical Studies

The serum activities of alanine aminotransferase (ALT) and aspartate aminotransferase (AST) were determined according to the method of Reitman and Frankel [22]. The serum alkaline phosphatase activity was measured according to the modified method of Moss [23].

The serum total protein levels were estimated according to Grant [24]. The serum albumin level was evaluated according to Doumas et al. [25]. According to Doumas and Biggs [26], the serum globulins levels were calculated mathematically by subtracting albumin values from total protein values.

The total serum lipids, total cholesterol, triglyceride, and high-density lipoprotein (HDL-c) were estimated according to the methods of Zöllner and Kirsch [27], Roeschlau et al. [28], McGowan et al. [29], and Young [30], respectively. Low-density lipoprotein (LDL-c) and very-low-density lipoprotein cholesterol (VLDL-C) were calculated mathematically according to the following relationship described by Friedewald et al. [31].

### 2.5. Detection of DNA Damage

The liver DNA damage was measured using a single-cell gel electrophoresis technique (also known as comet assay) as previously defined by Singh et al. [32]. Comet assay is a quick, accurate, and simple method for detecting DNA damage. In this method, 0.5 g of crushed samples were transferred to 1 mL ice-cold PBS. This suspension was stirred for 5 min and filtered. The cell suspension (100 μL) was mixed with 600 μL of low melting agarose (0.8% in PBS). A 100 µL of this mixture was spread on precoated slides. The coated slides were immersed in lysis buffer (0.045 mol/L Tris/Borate/EDTA (TBE), pH 8.4, containing 2.5% sodium dodecyl sulfate (SDS)) for 15 min. The electrophoresis conditions were 2 V/cm for 2 min and 100 mA. The slides were then washed 3 times, for 5 min each, with neutralization buffer (0.4 Mol/L Tris (pH 7.5)). Finally, the slides were stained with 50 AL of ethidium bromide (2 mg/mL) and covered with a coverslip.

The DNA fragment migration patterns of 100 cells at 400 magnifications with the Optika Axioscope fluorescence microscope were calculated for each dose level. The length of DNA migration (tail length) on PX was calculated for each cell from the center of the nucleus to the termination of the tail. By calculating the total intensity (fluorescence) in the cells, which was taken as 100%, the DNA% in the tail was determined, deciding what percentage of this total intensity corresponded to the intensity only measured in the tail. The tail moment was expressed in arbitrary units. Although any image analysis device may be sufficient for SCGE data quantification, Comet 5 image analysis software developed by Kinetic Imaging Ltd. (Liverpool, UK) linked to a CCD camera has been used to determine the degree of quantitative and qualitative DNA damage in the cells by measuring the length of DNA migration and the % of migrated DNA. Finally, the program calculated the tail moment. Generally, 100 randomly selected cells are analyzed per sample.

### 2.6. Histopathological Investigations

Samples were taken from the liver of euthanized chicks by manual cervical dislocation and fixed in formalin of 10%. The samples preserved with formalin are dehydrated and embedded in paraffin. Five-micron-thick paraffin slices were set and stained with hematoxylin and eosin (H&E) and inspected microscopically [33].

### 2.7. Statistical Analysis

Data were analyzed with a one-way analysis of variance (ANOVA) using the GLM procedure in SPSS (SPSS Inc., Chicago, IL, USA) after Shapiro–Wilk’s test was used to verify the normality and Levene’s test was used to verify homogeneity of variance components between experimental treatments. Duncan’s test was used to compare the differences between the means at 5% probability [34]. Variation in the data was expressed as mean ± SD, and the significance level was set at *p* < 0.05.

## 3. Results

### 3.1. Clinical Signs and Body Performance

No clinical signs or mortality were found in all supplemented birds (single or combined Vit. C and Vit. E addition) during the experimental period. The CuSO_4_-intoxicated group showed mild diarrhea (few cases), decreased appetite, and pale comb.

As presented in Table 2, the BW, BWG, and feed intake of broilers were significantly declined in all groups from the 2^nd^ week till the termination of the experiment (6th week) compared to the CON group (*p* < 0.05). However, these changes were minimized in birds supplemented with vitamins C and E compared with CuSO_4_-intoxicated group. The FCR was significantly higher in CuSO_4_-intoxicated broilers at the 2nd to 6th week of age compared to the control group (*p* < 0.05). Comparatively, with the CON group, the FCR was insignificantly different in birds fed diets supplemented with vitamin C, and E singly or in combination from the 3rd week of age and went back to near average control values at the 6th week (*p* < 0.05).

### 3.2. Serum Levels of Liver Biomarkers

As shown in Table 3, Table 4, Table 5 and Table 6, CuSO_4_ induced hepatotoxicity as reflected statistically (*p* < 0.05) by increased serum activities of ALT, AST, and ALP, whereas serum TP, albumin, globulins, TG, TC, LDL-C, VLDL-C, and HDL-C levels were reduced at 3rd and 6th week compared to CON (*p* ≤ 0.05). The decrease in serum albumin and HDL-C was only in the 6th week period.

On the other hand, co-supplementation of copper with Vit. C and Vit. E significantly lowered the serum AST, ALT, and ALP and increased serum TP, albumin, globulins, TG, TC, LDL-C, VLDL-C, and HDL-C levels in the CuSO_4_ + Vit. C and CuSO_4_ + Vit. E groups compared with CuSO_4_-induced hepatotoxicity group. However, co-administration of CuSO_4_ + Vit. C + Vit. E restored liver biomarkers’ changes near-normal control values.

### 3.3. DNA Damage

**Figure 1:**
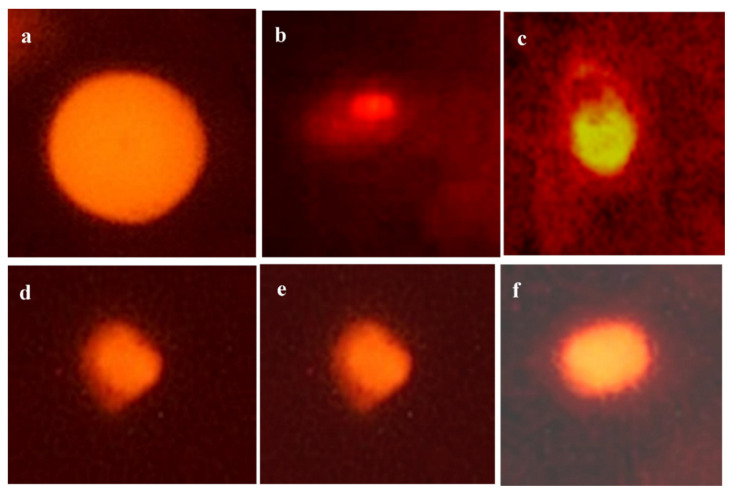
Electrophoresis of hepatic DNA samples of chicken from all experimental groups, using 1.2% ethidium bromide-stained agarose (×400). (**a**)**:** The control group showed no DNA damage, as demonstrated by the lack of DNA fragment migration away from the nucleus core. (**b**,**c**): CuSO4 group showed a high degree of DNA damage, with a significantly reduced nucleus core and a large cloud of DNA fragments migrating away from the core, forming the characteristic comet tail. (**d**): CuSO_4_ + Vit. C group showed a moderate degree of DNA damage. (**e**): CuSO_4_ + Vit. E group showed a reasonable degree of DNA damage. (**f**): CuSO_4_ + Vit C + Vit E group showed a minimal degree of DNA damage.

The data in Table 7 and Table 8 and Figure 1 revealed that CuSO_4_ intoxication significantly elevated the comet %, %DNA in the tail, tail moment, olive tail moment, and tail length at the end of the 3rd and 6th week parallel to CON (*p* < 0.05). On the contrary, co-administration of CuSO_4_ + Vit. C, CuSO_4_ + Vit. E, or their combination (CuSO_4_ + Vit. C + Vit. E) to birds ensued in an enhancement in the results of comet assay performance, showing a substantial decrease in previous parameters relative to the CuSO_4_-intoxicated group but did not return to values of standard control. It was evident that the vitamin combination led to a significant decrease in comparison to the CuSO_4_-intoxicated group.

### 3.4. Histopathological Findings

**Figure 2:**
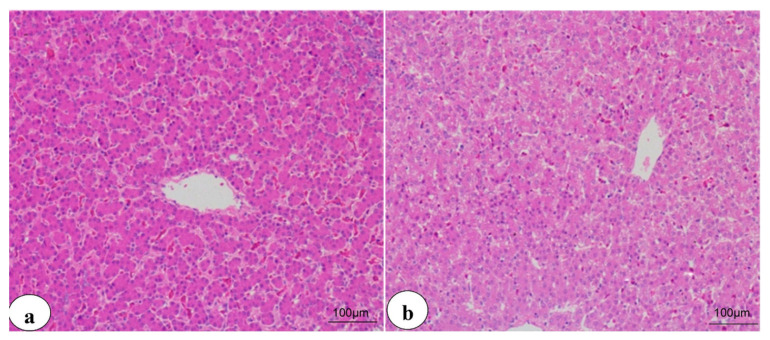
Photomicrographs of liver sections from chickens of the control group showed normal histopathological structures at the end of the 3rd week (**a**) and 6th week (**b**).

**Figure 3:**
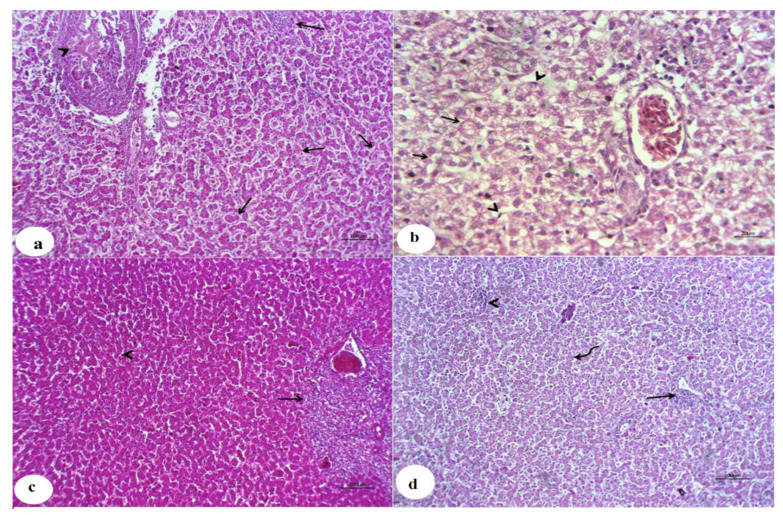
Photomicrographs of liver sections for chickens of all experimental groups at the end of the 3rd week. (**a**): Liver of CuSO_4_-intoxicated chicken showed hyperplastic and necrotic biliary epithelium (arrow) with various degenerative and necrotic changes in the hepatic cells (arrowhead). (**b**): Liver of CuSO_4_ + Vit. C group showed moderate swelling of hepatic cells (arrow) and hyperplastic Kupffer cells (arrowhead). (**c**): Liver of CuSO_4_ + Vit. E group showed lymphocytic portal aggregation (arrow) within apparently normal hepatic parenchyma (arrowhead). (**d**): Liver of CuSO_4_ + Vit. C + Vit. E group showed little portal (arrow) and interstitial lymphocytic aggregations within apparently normal hepatic parenchyma (arrowhead).

**Figure 4:**
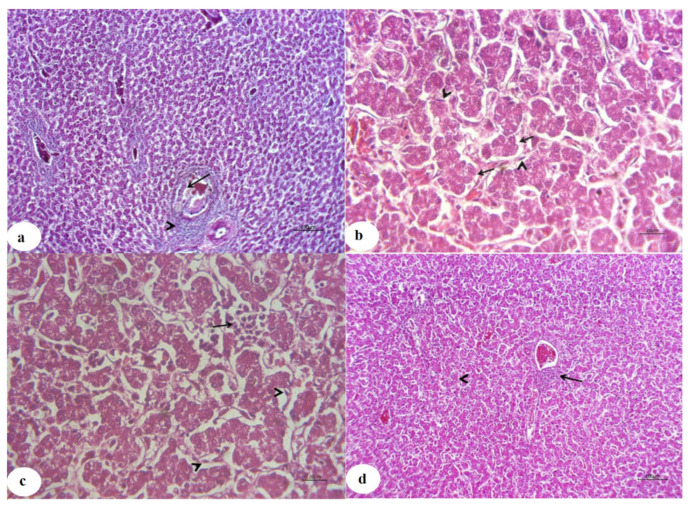
Photomicrographs of liver sections for chickens of all experimental groups at the end of the 6th week. (**a**): Liver of CuSO_4_-intoxicated chicken showed cholestasis, necrotic bile duct epithelia (arrow), besides portal lymphocytic aggregation and fibroblast proliferation (arrowhead). (**b**): Liver of CuSO_4_ + Vit. C supplemented chicken showed partially contracted hepatic cells (arrow), proliferative Kupffer cells, and dilated sinusoids (arrowhead). (**c**): Liver of CuSO_4_ + Vit. E supplemented chicken showed intense hyperplasia of Kupffer cells (arrowhead) and cloudy swelling of hepatic cells with a few lymphocytic aggregations (arrow). (**d**): Liver of CuSO_4_ + Vit. C + Vit. E supplemented chicken showed mild portal lymphocytic aggregation (arrow) and normal hepatic parenchyma (arrowhead) with dilated blood vessels.

The livers of the control chicken showed typical histological arrangement of hepatic lobules at the 3rd and 6th weeks (Figure 2a,b), respectively. In contrast, the livers of chicken from the CuSO_4_-intoxicated group displayed hyperplastic and necrotic biliary epithelium with various degenerative and necrotic changes at the third week (Figure 3a). Additionally, cholestasis, necrotic bile duct epithelia, besides lymphocytic portal aggregation and fibroblast proliferation, were encountered at the 6th week (Figure 4a). Liver from chicken of the CuSO_4_ + Vit. C group showed moderate enlargement of hepatic cells and hyperplastic Kupffer cells at the 3rd week (Figure 3b) and partially contracted hepatic cells proliferative Kupffer cells and dilated sinusoids at the 6th week (Figure 4b). Liver from chicken of the CuSO_4_ +Vit. E supplemented group revealed lymphocytic portal aggregations within apparently normal hepatic parenchyma at the third week (Figure 3c), while intense hyperplasia of Kupffer cells and cloudy swelling of hepatic cells with a few lymphocytic aggregations were seen at the 6th week (Figure 4c). Liver of the CuSO_4_ + Vit. C + Vit. E group displayed little portal and interstitial lymphocytic aggregations with apparently normal hepatic parenchyma at the third week (Figure 3d), mild portal lymphocytic aggregation, normal hepatic parenchyma, and dilated blood vessels were observed at the 6th week (Figure 4d).

## 4. Discussion

Although the level of copper up to 100 to 200 mg/kg as CuSO_4_ improves growth performance in the broilers [9,35,36,37], incompatible effects such as poor feed intake, decrease in body weight, and hematobiochemical changes at higher doses of copper have been reported [38,39,40]. In this study, no clinical signs or mortality were perceived during the experimental period in Vit. C and E supplemented groups, while CuSO_4_-intoxicated birds showed mild diarrhea, anorexia, and weight reduction. This was synchronized with Luo et al. [41], who reported zero mortality in male chicks supplemented with 300 and 450 mg/kg CuSO_4_ for 21 days. Other studies reported no significant differences in mortality % of broiler chickens among the control and other groups given 0, 10, 25, 50, 125, 250, and 500 CuSO_4_/kg diet for 35 days [42] or 100, 200, and 400 mg CuSO_4_/kg diet for 42 days [43].

The exiting investigation showed that copper sulfate had a toxic effect, as exhibited by a statistical reduction in growth efficiency parameters (BW, BWG, and FI), increasing the FCR over the entire growth-out period (days 1–42) in CuSO_4_-intoxicated birds. These findings may be attributed to falling in the feed intake and utilization as a result of GIT disturbance caused by a highly toxic dose of Cu or owing to the embarrassment of the satiety center by Cu, leading to loss of interest in feeding [44]. Additionally, the reduction in feed consumption could be due to an anorexic effect of CuSO_4_ on chickens [41,45]. Our results were consistent with Luo et al. [41], Cinar et al. [17], Abduljaleel [46], Scott et al. [36], and Zhou et al. [47]. Previous studies recorded different outcomes such as unchanged BW and FCR in chicks fed up to 400 mg Cu/kg [48] and improved live weight gain in broiler chick supplemented with 400 mg/kg feed copper sulfate for 6 weeks [43]. The reason for variances between our experimental results and others may be concurrent with the varieties in the CuSO4 particle size, stain, and age of birds, experimental periods, and the dose of copper between earlier and current experiments. Furthermore, the difference between the effects of Cu levels on the growth performance of raising poultry indicates that the bioavailability of Cu can differ. This was supported by other literature stating that copper at sufficient dietary levels has favorable impacts, but severe toxic effects as a result of extra copper are well established [49,50]. The study of Morsy et al. [51] investigated the oral administration of copper oxide nanoparticles (CuO-NPs) at doses 5 mg/kg and 15 mg/kg BW in broiler chickens. They showed a notable decrease in weight gain, FCR in a level-dependent manner. The growth parameters were upgraded meaningfully in CuSO_4_ + Vit. C and CuSO_4_ + Vit. E-supplemented groups compared to CuSO_4_-intoxicated group; however, better results have been found in birds taking both Vit. C and Vit E. Many reports showed better performance by feeding Vit. C or Vit. E to broiler chicks [17,46,52,53,54] or fish [55,56,57,58,59]. The improvements in performance characteristics in vitamin-supplemented groups could be attributable to their antioxidant effects that protect the bird from the oxidative stress caused by Cu exposure [60,61] or the role of vitamins as an immune stimulant [62].

Birds supplemented with excess CuSO_4_ revealed an elevation in serum transaminases (ALT and AST) and ALP activities correlated with diminished serum TP, albumin, and globulins levels equated to the control group, suggesting significant liver damage (hepatotoxicity), which was established by hyperplastic and necrotic biliary epithelium, hepatic degeneration, and necrosis in the histopathological findings. The increased transaminase activities in the CuSO_4_-intoxicated group may be associated with the influence of Cu on the liver and kidney, so releasing their intracellular enzymes to circulation [63] or due to the cytotoxic effect of Cu, resulting in lipid peroxidation indicating the hepatotoxic effect of Cu [64]. In addition, Kumar et al. [43] determined that a higher level of copper accumulation may have injured the liver to rise these enzymes. Abundant studies indicate that copper can be metabolized in hepatic tissues and converted by glutathione (GSH) into metallothionein; Therefore, copper excess is got, and the immediate results of GSH are depleted of enhanced cytotoxicity [65].The finding of high serum enzymes in Cu-intoxicated broilers agreed with Yigit et al. [49], who stated that copper produced changes in the liver transaminases of broilers. As a result of CuSO_4_ addition, the upsurge in ALP activity might be recognized for liver, intestine, kidney, and to some degree, bile duct injuries, especially liver cell membrane, which appears to act as a stimulus to increase the synthesis of this enzyme or may be due to hepatic or bile duct cholestasis causing enzyme regurgitation back into the bloodstream [66].

The reported hypoproteinemia in this inquiry may be due to impairment protein synthesis or the functional deterioration of the liver and excessive loss of protein caused by nephrosis [67] or could be explained due to the oxidative stress of copper on the liver and kidney tissue [11,39]. Since albumin has unique copper ion binding sites and carries dietary copper to the liver [68], the distinguished drop in the serum albumin levels may be due to the decrease in the synthesis of hepatic albumin in the CuSO_4_ group. Contrary to our results, Almansour [69] reported intensification in serum protein levels in copper-fed quail. Cinar et al. [17] displayed no alteration in plasma proteins of 300 mg/kg diet copper-fed bird. Dietary addition of Vit. C and Vit. E alone or in combination with CuSO_4_-intoxicated birds significantly ameliorated the previous changes in liver enzymes, demonstrating that these vitamins have potential protecting effects on cell membranes, thus preventing enzyme leak into the blood [17,70]. Better results were evident with vitamin supplementation than each alone because some liver biomarkers were returned near the average control values. These results were in synchronization with those attained by [71], Abou-Kassem et al. [72], Mashkoor et al. [73], and Hashem et al. [20]. The hepatoprotective influence of vitamin C or E is associated with their antioxidant properties [74]. Additionally, ascorbic acid causes its association with heavy metals, which leads to reduced tissue oxidative stress and restored AST, ALT, ALP and LDH levels [75]. Besides, vitamins C and E may preserve the hepatic cellular membrane and protect hepatocyte from copper’s toxic effects, minimizing the enzyme’s leakage into the bloodstream [70]. In dissimilarity, supplementing cadmium-intoxicated broilers with vitamins C and E did not recover transaminase activities [76].

Co-supplementation of copper with Vit. C and Vit. E alone or in combination elevated serum TP, albumin, globulins. The enhancement in the protein profile in Cu-exposed birds fed with Vit. C and Vit. E alone or in combination may be due to the immunostimulant effect [77] or due to impairments of the copper uptake and utilization [17] by dietary supplement of Vit. C or Vit. E. Imik et al. [78] reported a high total protein concentration in blood of quail supplemented vitamin E and C in the diet. Therefore, only the co-administration of copper with both vitamins had a protecting effect to hepatotoxicity caused by CuSO_4_.

Regarding lipogram analysis, CuSO_4_ administration causes dyslipidemia, evidenced by a numerical reduction in serum TG, TC, LDL-C, and VLDL-C levels; however, serum HDL-C did not display any change in the third week but significantly dropped at 6th week in CuSO_4_—intoxicated birds compared with the control group. Our findings were consistent with Bakalli et al. [79] and Idowu et al. [71]. Moreover, Wu et al. [80] noticed significantly lessened serum cholesterol and LDL-C levels in broilers fed 3 sources of copper (copper methionate, tribasic copper chloride, and copper sulfate) in the diet. The reduction in plasma cholesterol and triacylglycerol in the blood of Cu-exposed chickens is due to fall cholesterol synthesis, high degradation, or excretion rates [81]. The excess level of Cu supplementation to diet either declines GSH that diminished stimulation of HMG-CoA reductase activity resulted in the reduced synthesis of cholesterol [82], or lead to changes in lipid metabolism, which result in decreasing plasma lipid, 17 beta-estradiol, and hepatic lipogenic enzyme activity [83]. The reduction in LDL level was related to copper toxicity because Cu is an effective catalyst of LDL-C oxidation to an atherogenic form [84] or alkoxyl radicals [85]. The reduction in HDL-C is due to hypocholesterolemia and hypoproteinemia in this inquiry, as more than 40% of HDL-C value represents cholesterol value and the remaining proteins [86]. The same findings were obtained by El-Hady and Mohamed [87] in broiler chickens with dietary supplementation of CuSO_4_ at levels 50 and 100 ppm for 5 weeks. However, earlier studies exposed different outcomes, for example, rise of total lipid, cholesterol, and LDL-C with no variation HDL-C level in Cu-exposed quail [69] or no alteration in plasma total cholesterol levels in broilers [17]. Additionally, Jegede et al. [35] informed a decrease in plasma triglycerides and cholesterol in Arbor-Acre unsexed broilers fed CuSO_4_ or copper proteinate at concentrations of 50, 100, or 150 mg/kg diet for 56 days. The breed, dietary components, and the investigational strategy and methodology can explain these differences.

Dietary supplementations of CuSO_4_-intoxicated birds with Vit. C and Vit. E alone or in combination reduce the opposing effects of copper on lipid profile. Treatment of Cu toxicity with Vit. C and Vit. E combination is more effective than using each one separately. This can be due to the ability of the vitamin C to replenish the vitamin E re-mobilizes the free radicals associated with it [88]. The combination of vitamins protects lipid structures against peroxidation [70]. Vitamin C guards against Cu toxicity by preventing excess Cu absorption by minimizing copper absorption from the intestine by reducing soluble Cu levels in the small intestine [89,90]. Besides, Vit. E can prevent cholesterol-related endothelial dysfunction, preventing functional impairment induced by ROS [13].

Although the micronucleus assay has been useful and its utility should be considered [91], the comet assay is a more sensitive method for assessing genotoxicity [92]. The true comet assay was used to test for genotoxic agents [93]. Collaboration with others is a sensitive and fast method for distinguishing DNA damage produced by trace metals, such as copper [94]. 

Data from this research showed that CuSO_4_ has the genotoxic ability to interact with DNA and induce mammalian cell alterations specified by elevation in comet %, DNA % in the tail, tail length, tail moment, and olive tail moment. Similarly, Banu et al. [95] indicated significant DNA damage with a decrease in mean comet tail-length after adding CuSO_4_. Copper-induced DNA damage may or may not be constrained at low copper concentrations, as it is closely bound to storage or transport proteins (eg. ceruloplasmin) and thus not available for oxidative reactions [96], but at high concentrations, free Cu can have an enormous genotoxic effect [91]. Free Cu causes ROS and multiple types of DNA damage, such as base alteration and DNA strand breaks, which can cause severe cell death [97]. A copper-induced high ROS production consequence in oxidative destruction to a single DNA base and sugar phosphate and breaks DNA strands [98]. Additionally, copper reduces DNA-binding cell viability, resulting in cell death [99].

The current study revealed that supplementing of CuSO_4_-intoxicated chickens with Vit. C and Vit. E alone or in combination exert a partial genoprotective effect against DNA damage induced by excessive concentrations of copper, which was proved by the decrease of comet%, tail length, and moment. A combination of vitamins is more effective in reducing the genotoxic effects of copper than using each vitamin alone. The genoprotective effect of Vit. C and Vit. E could act as free radical scavengers and antioxidants [100]. Jiraungkoorskul and Sahaphong [101] demonstrated that ascorbic acid reduces genotoxicity in fish induced by copper. Assy et al. [102] exhibited a defending effect of Vit. E (100 mg/kg diet) against DNA damage in rats with CuO nanoparticles toxicity (250 mg/kg diet). Our outcomes are in settlement with a new study reporting potential anticipatory effects of dietary antioxidants, including vitamin E, ascorbic acid, phytosterols, polyphenols, and medicinal plants extracts against vanadium-induced DNA damage [103]. Another study showed that curcumin supplementation reduced genomic and cellular DNA damage in mice exposed to 390 ppm Cu [91]. Morsy et al. [51] reported increased DNA fragmentation percent and microscopic recording in various inspected organs of chickens received CuO-NPs.

Histopathological check established the results of CuSO_4_-intoxicated chickens in the existing inquest and it is in coordination with the earlier report of Oguz et al. [38], Shahzad et al. [40], Wang et al. [11] in chickens and Baruah et al. [104] in ducks with copper toxicity. Similarly, other studies exhibited a significant inflammatory cell infiltration and hepatocyte vacuolar degeneration [105], severe microscopic changes, including vacuolar degeneration, local tissue necrosis, and blurred hepatic lobules in birds-supplemented with 300 mg Cu/kg diet [47]. Morsy et al. [51] observed histopathological changes in chickens getting CuO-NPs with some differences in its severity. Our results differ from other findings that showed that birds fed a high level of copper diet did not alter any histological tissue [106]. These inappropriate associations may be attributed to the variance in the types of test subjects, experimental animals, and test duration. Long-term exposure of birds to high levels of copper, dangerous effects may occur [47]. However, single or combined addition with Vit. C and Vit. E decreased the histopathological alterations with apparently typical liver architecture in some cases. Other findings presented that the addition of vitamins C and E in the feed has successfully offset arsenite’s toxic effects in broiler chicken [107].

## 5. Conclusions

The obtained findings could conclude that long-term exposure to CuSO_4_ caused significant alterations in the liver evaluation biomarkers, genotoxicity, and histopathology. Dietary addition of vitamins C and E reduced the harmful effects induced by CuSO_4_, especially with their combination, which caused an improvement in the growth performance; returned the biochemical parameters in close to average values, with subsidence the histopathological changes and DNA degeneration. Overall, the protective roles of vitamins C and E with their synergistic action against the toxic effects of Cu are seen in our research, but further studies are still needed to understand the full potential of vitamins.

## Figures and Tables

**Table 1:** Proximate and chemical composition of the basal diets (%).

Ingredients	Starter Stage (1–10 Day)	Grower Stage (11–22 Day)	Finisher Stage (23–42 Day)
Soybean meal, 48%	34.66	28.2	25
Yellow corn	58	62	63.5
Corn gluten, 60%	1.5	3	3
Wheat bran	--	1.1	1.8
Soy oil	2	2	3.26
Calcium dibasic phosphate	1.8	1.7	1.5
Calcium carbonate	1	1	1
Premix *	0.3	0.3	0.3
Lysine, Hcl, 78%	0.16	0.16	0.13
Common salt	0.3	0.3	0.3
DL-Methionine, 98%	0.18	0.14	0.11
Anti-mycotoxin	0.1	0.1	0.1
Chemical composition (%)			
ME, Kcal/Kg	3047.51	3090.11	3178.56
Crude protein	22.16	20.42	19.09
Crude fiber	2.63	2.64	2.65
Fat	4.51	4.63	5.89
Calcium	0.97	0.94	0.88
Available P	0.48	0.45	0.41
Methionine	0.55	0.48	0.46
Lysine	1.37	1.20	1.09

* Premix per kg of diet: vitamin A, 1 500 IU; vitamin D3, 200 IU; vitamin E, 10 mg; vitamin K3, 0.5 mg; thiamine, 1.8 mg; riboflavin, 3.6 mg; pantothenic cid, 10 mg; folic acid, 0.55 mg; pyridoxine, 3.5 mg; niacin, 35 mg; cobalamin, 0.01 mg; biotin, 0.15 mg; Fe, 80 mg; Cu, 8 mg; Mn, 60 mg; Zn, 40 mg; I, 0.35 mg; Se, 0.15 mg. ME: metabolizable energy; P: phosphorus.

**Table 2:** Effect of single or combined supplementation of vitamin C and E on the growth performance of CuSO_4_-intoxicated broiler chickens (Mean ± SD).

Parameters	Weeks	CON	CuSO_4_	CuSO_4_ + Vit. C	CuSO_4_ + Vit. E	CuSO_4_ + Vit. C + Vit. E	*p*-Value
Int. BW		43.8 ± 4.15	43.8 ± 4.14	44.0 ± 2.23	43.8 ± 2.77	43.4 ± 2.30	0.241
BW (g/bird)	1st week	159.0 ± 4.18	152.2 ± 6.83	153.0 ± 5.70	154.0 ± 5.47	156.0 ± 4.18	0.120
	2nd week	430 ± 16.95 ^a^	377 ± 20.73 ^c^	389 ± 7.41 ^c^	399.4 ± 11.33 ^bc^	422 ± 12.04 ^b^	0.001
	3rd week	847 ± 24.64 ^a^	712 ± 23.87 ^d^	762 ± 09.08 ^c^	783 ± 09.08 ^c^	814 ± 21.03 ^b^	0.001
	4th week	1281 ± 32.09 ^a^	1060 ± 39.21 ^d^	1149 ± 27.70 ^c^	1178 ± 16.43 ^c^	1221 ± 28.15 ^b^	0.009
	5th week	1818 ± 44.24 ^a^	1500 ± 18.02 ^e^	1614 ± 35.07 ^d^	1663 ± 25.64 ^c^	1716 ± 42.48 ^b^	0.008
	6th week	2430 ± 63.54 ^a^	2000 ± 25.49 ^d^	2136 ± 42.92 ^c^	2198 ± 44.38 ^c^	2271 ± 68.32 ^b^	0.001
BWG (g/bird)	1st week	115.2 ± 5.00	108.4 ± 7.56	109.0 ± 6.51	110.2 ± 3.27	112.6 ± 4.87	0.542
	2nd week	271 ± 16.35 ^a^	224.8 ± 37.92 ^c^	236 ± 8.94 ^bc^	250 ± 15.00 ^ab^	266 ± 12.44 ^b^	0.000
	3rd week	417 ± 22.09 ^a^	335 ± 21.21 ^c^	373 ± 6.70 ^b^	383.6 ± 15.56 ^b^	392 ± 12.54 ^b^	0.000
	4th week	434 ± 15.57 ^a^	348 ± 30.53 ^c^	387 ± 30.12 ^b^	395 ± 22.52 ^b^	407 ± 26.12 ^ab^	0.001
	5th week	537 ± 33.27 ^a^	440 ± 27.15 ^c^	465 ± 21.50 ^bc^	485 ± 20.30 ^b^	495 ± 16.95 ^b^	0.001
	6th week	612 ± 23.87 ^a^	500 ± 26.45 ^c^	522 ± 23.623 ^bc^	535 ± 26.45 ^bc^	555 ± 32.78 ^b^	0.000
Feed intake (g/bird)	1st week	144.5 ± 6.15	139.85 ± 7.25	140 ± 10.12	141 ± 9.20	142.4 ± 5.15	0.270
	2nd week	370 ± 20.15	325 ± 24.26	333 ± 15.14	348 ± 17.24	365 ± 35.10	0.752
	3rd week	583 ± 35.00 ^a^	525 ± 26.14 ^c^	537 ± 19.15 ^b^	548 ± 36.09 ^b^	557 ± 24.10 ^ab^	0.000
	4th week	700 ± 27.12 ^a^	625 ± 15.00 ^c^	635 ± 20.8 ^b^	650 ± 27.95 ^b^	660 ± 37.00 ^ab^	0.000
	5th week	900 ± 33.14 ^a^	810 ± 31.13 ^c^	825 ± 16.9 ^b^	845 ± 32.09 ^b^	855 ± 28.4 ^b^	0.009
	6th week	1070 ± 55.12 ^a^	950 ± 34.24 ^c^	965 ± 29.9 ^b^	977 ± 16.7 ^b^	999 ± 45.74 ^ab^	0.001
FCR	1st week	1.25 ± 0.05	1.29 ± 0.09	1.28 ± 0.07	1.27 ± 0.03	1.26 ± 0.05	0.483
	2nd week	1.36 ± 0.08	1.45 ± 0.12	1.41 ± 0.05	1.39 ± 0.08	1.39 ± 0.06	0.335
	3rd week	1.40 ± 0.06 ^b^	1.57 ± 0.06 ^a^	1.44 ± 0.03 ^b^	1.43 ± 0.05 ^b^	1.42 ± 0.04 ^b^	0.000
	4th week	1.61 ± 0.05 ^b^	1.79 ± 0.17 ^a^	1.65 ± 0.13 ^ab^	1.64 ± 0.09 ^ab^	1.62 ± 0.13 ^ab^	0.007
	5th week	1.67 ± 0.10 ^b^	1.83 ± 0.08 ^a^	1.77 ± 0. 08 ^ab^	1.74 ± 0.07 ^ab^	1.71 ± 0.06 ^ab^	0.000
	6th week	1.74 ± 0.06 ^b^	1.90 ± 0.10 ^a^	1.84 ± 0.08 ^ab^	1.82 ± 0.09 ^ab^	1.80 ± 0.08 ^ab^	0.000

^a, b, c, d^ Means carrying different superscripts are significantly different at *p* < 0.05. BW: body weight, BWG: body weight gain, FCR: feed conversion ratio.

**Table 3:** Effect of single or combined supplementation of vitamin C and E on broiler chickens’ liver enzyme activities and protein profile at the end of the 3rd week (Mean ± SD, *n* = 5).

Parameters	CON	CuSO_4_	CuSO_4_ + Vit. C	CuSO_4_ + Vit. E	CuSO_4_ + Vit. C + Vit. E	*p*-Value
ALT (U/L)	9.30 ± 0.99 ^c^	21.60 ± 6.14 ^a^	16.60 ± 4.41 ^b^	15.60 ± 4.41 ^b^	13.10 ± 3.29 ^bc^	0.001
AST (U/L)	150.5 ± 10.9 ^d^	193.7 ± 9.2 ^a^	177.9 ± 5.6 ^b^	171.8 ± 7.4 ^bc^	162.6 ± 13.9 ^cd^	0.001
ALP (U/L)	620 ± 71.2 ^c^	727 ± 58.3 ^a^	711 ± 99.2 ^a^	702 ± 95.1 ^a^	689 ± 57.5 ^b^	0.001
TP (g/dL)	3.69 ± 0.20 ^a^	3.33 ± 0.18 ^b^	3.52 ± 0.07 ^b^	3.65 ± 0.12 ^a^	3.67 ± 0.32 ^a^	0.000
Albumin (g/dL)	1.99 ± 0.13	1.83 ± 0.15	1.84 ± 0.15	1.94 ± 0.16	1.95 ± 0.15	0.453
Globulins (g/dL)	1.70 ± 0.08 ^a^	1.50 ± 0.09 ^b^	1.68 ± 0.12 ^a^	1.71 ± 0.19 ^a^	1.72 ± 0.13 ^a^	0.000

^a, b, c, d^ Means carrying different superscripts are significantly different at *p* < 0.05. ALT: alanine aminotransferase, AST: aspartate aminotransferase, ALP: alkaline phosphatase, TP: total proteins.

**Table 4:** Effect of single or combined supplementation of vitamin C and E on broiler chickens’ liver enzyme activities and protein profile at the end of the 6th week (Mean ± SD, *n* = 5).

Parameters	CON	CuSO_4_	CuSO_4_ + Vit. C	CuSO_4_ + Vit. E	CuSO_4_ + Vit. C + Vit. E	*p*-Value
ALT (U/L)	11.56 ± 4.86 ^c^	24.42 ± 5.00 ^a^	18.42 ± 3.35 ^b^	16.87 ± 3.72 ^b^	15.25 ± 3.12 ^bc^	0.001
AST (U/L)	160.5 ± 9.1 ^d^	195.7 ± 7.8 ^a^	182.3 ± 5.5 ^b^	177.8 ± 5.4 ^bc^	169.6 ± 7.6 ^cd^	0.001
ALP (U/L)	580 ± 31.1 ^b^	660.0 ± 54 ^a^	628 ± 44 ^ab^	616 ± 55 ^ab^	578 ± 30 ^b^	0.000
TP (g/dL)	4.33 ± 0.42 ^a^	3.01 ± 0.18 ^b^	4.15 ± 0.36 ^a^	4.17 ± 0.40 ^a^	4.30 ± 0.33 ^a^	0.000
Albumin (g/dL)	2.48 ± 0.13 ^a^	2.01 ± 0.24 ^b^	2.27 ± 0.19 ^ab^	2.28 ± 0.18 ^ab^	2.30 ± 0.20 ^a^	0.011
Globulins (g/dL)	1.85 ± 0.69 ^a^	0.9 ± 0.18 ^b^	1.88 ± 0.25 ^a^	1.89 ± 0.24 ^a^	2.00 ± 0.32 ^a^	0.000

^a, b, c, d^ Means carrying different superscripts are significantly different at *p* < 0.05. ALT: alanine aminotransferase, AST: aspartate aminotransferase, ALP: alkaline phosphatase, TP: total proteins.

**Table 5:** Effect of single or combined supplementation of vitamin C and E on the lipid profile of broiler chickens at the end of the 3rd week (Mean ± SD, *n* = 5).

Parameters	CON	CuSO_4_	CuSO_4_ + Vit. C	CuSO_4_ + Vit. E	CuSO_4_ + Vit. C + Vit. E	*p*-Value
TG (mg/dL)	65.03 ± 4.74 ^a^	48.14 ± 7.33 ^c^	50.90 ± 5.57 ^bc^	53.64 ± 3.49 ^bc^	60.12 ± 5.91 ^ab^	0.000
TC (mg/dL)	155.82 ± 2.94 ^a^	126.79 ± 11.00 ^c^	130.48 ± 6.75 ^c^	138.92 ± 5.51 ^bc^	148.48 ± 5.78 ^ab^	0.000
LDL-C (mg/dL)	89.52 ± 3.19 ^a^	70.65 ± 7.74 ^c^	72.86 ± 5.53 ^c^	78.56 ± 1.95 ^bc^	84.66 ± 3.94 ^ab^	0.001
VLDL-C (mg/dL)	13.00 ± 0.98 ^a^	9.62 ± 1.46 ^c^	10.17 ± 1.11 ^bc^	10.72 ± 0.69 ^bc^	12.02 ± 1.18 ^ab^	0.000
HDL-C (mg/dL)	53.29 ± 3.49	46.51 ± 4.79	47.43 ± 2.60	49.78 ± 5.73	52.12 ± 7.23	0.701

^a, b, c, d^ Means carrying different superscripts are significantly different at *p* < 0.05. TG: triglycerides, TC: total cholesterol, LDL-C: low-density lipoprotein-cholesterol, VLDL-C: very low-density lipoprotein-cholesterol, HLDL-C: high-density lipoprotein-cholesterol.

**Table 6:** Effect of single or combined supplementation of vitamin C and E on the lipid profile of broiler chickens at the end of the 6th week (Mean ± SD, *n* = 5).

Parameters	CON	CuSO_4_	CuSO_4_ + Vit. C	CuSO_4_ + Vit. E	CuSO_4_ + Vit. C + Vit. E	*p*-Value
TG (mg/dL)	67.96 ± 2.32 ^a^	50.58 ± 5.42 ^c^	57.34 ± 1.85 ^bc^	58.23 ± 3.02 ^bc^	62.43 ± 6.19 ^ab^	0.000
TC (mg/dL)	166.68 ± 7.77 ^a^	130.86 ± 4.15 ^b^	135.95 ± 4.51 ^b^	141.36 ± 7.65 ^b^	157.40 ± 5.92 ^a^	0.000
LDL-C (mg/dL)	98.75 ± 6.50 ^a^	74.52 ± 5.36 ^c^	79.38 ± 4.59 ^c^	81.88 ± 4.50 ^bc^	91.51 ± 7.34 ^ab^	0.000
VLDL-C (mg/dL)	13.58 ± 0.46 ^a^	10.11 ± 1.08 ^c^	11.46 ± 0.36 ^bc^	11.64 ± 0.60 ^bc^	12.48 ± 1.23 ^ab^	0.007
HDL-C (mg/dL)	54.33 ± 1.91 ^ab^	46.22 ± 3.1 ^b^	47.95 ± 4.07 ^b^	50.16 ± 8.55 ^bc^	53.41 ± 4.67 ^ab^	0.000

^a, b, c^ Means carrying different superscripts are significantly different at *p* < 0.05. TG: triglycerides, TC: total cholesterol, LDL-C: low-density lipoprotein-cholesterol, VLDL-C: very low-density lipoprotein-cholesterol, HLDL-C: high-density lipoprotein-cholesterol.

**Table 7:** Effect of single or combined supplementation of vitamin C and E on the DNA degradation indices in broiler chickens at the end of the 3rd week (Mean ± SD).

Parameters	CON	CuSO_4_	CuSO_4_ + Vit. C	CuSO_4_ + Vit. E	CuSO_4_ + Vit. C + Vit. E	*p*-Value
Comet %	13.38 ± 1.17 ^c^	21.19 ± 1.13 ^a^	19.38 ± 1.05 ^ab^	18.96 ± 2.00 ^ab^	17.50 ± 2.62 ^b^	0.000
DNA in tail %	1.09 ± 0.03 ^e^	3.14 ± 0.06 ^a^	2.47 ± 0.05 ^b^	1.90 ± 0.04 ^c^	1.34 ± 0.06 ^d^	0.009
Tail length (Pixel)	1.14 ± 0.02 ^c^	2.06 ± 0.09 ^a^	1.92 ± 0.03 ^b^	1.90 ± 0.03 ^b^	1.88 ± 0.02 ^b^	0.001
Tail moment (Arbitrary units)	0.01 ± 0.006 ^e^	0.06 ± 0.002 ^a^	0.04 ± 0.001 ^b^	0.03 ± 0.001 ^c^	0.02 ± 0.003 ^d^	0.001
Olive tail moment	0.15 ± 0.05 ^c^	0.35 ± 0.06 ^a^	0.31 ± 0.08 ^a^	0.25 ± 0.05 ^b^	0.27 ± 0.03 ^b^	0.000

^a, b, c, d, e^ Means carrying different superscripts are significantly different at *p* < 0.05. Values are expressed as % of total counts in each assay.

**Table 8:** Effect of single or combined supplementation of vitamin C and E on the DNA degradation indices in broiler chickens at the end of the 6th week (Mean ± SD).

Parameters	CON	CuSO_4_	CuSO_4_ + Vit. C	CuSO_4_ + Vit. E	CuSO_4_ + Vit. C + Vit. E	*p*-Value
Comet %	14.56 ± 1.69 ^c^	25.90 ± 2.14 ^a^	23.95 ± 1.50 ^ab^	23.81 ± 3.24 ^ab^	19.95 ± 2.95 ^b^	0.000
DNA in tail %	1.25 ± 0.03 ^e^	3. 47 ± 0.06 ^a^	2.77 ± 0.05 ^b^	2.07 ± 0.04 ^c^	1.38 ± 0.02 ^d^	0.000
Tail length (Pixel)	1.99 ± 0.08 ^e^	3.00 ± 0.10 ^a^	2.70 ± 0.13 ^b^	2.50 ± 0.10 ^bc^	2.27 ± 0.07 ^d^	0.000
Tail moment (Arbitrary units)	0.02 ± 0.009 ^e^	0.10 ± 0.017 ^a^	0.07 ± 0.022 ^b^	0.05 ± 0.017 ^c^	0.03 ± 0.014 ^d^	0.009
Olive tail moment	0.21 ± 0.03 ^c^	0.42 ± 0.03 ^a^	0.36 ± 0.06 ^b^	0.31 ± 0.02 ^b^	0.29 ± 0.01 ^b^	0.004

^a, b, c, d, e^ Means carrying different superscripts are significantly different at *p* < 0.05. Values are expressed as % of total counts in each assay.

## Data Availability

Data sharing not applicable.
